# The Adulterations of Milk

**Published:** 1863-08

**Authors:** 


### THE ADULTERATIONS OF MILK.

It is well known that much of the milk which is supplied in large towns is almost constantly more or less adulterated, and although the substances employed for the purpose are in most cases comparatively innoxious, it is much to be wished that some simple and efficient test of its genuineness and purity could be devised, capable of being applied by those who are unaccustomed to experiment.

The chief mode of adulteration practiced in this country consists in diluting the milk with water, and at the same time occasionally removing the cream. To correct the bluish color of the impoverished milk, it is said that a little annatto is sometimes added. Milk has been occasionally found adulterated with gum, flour, and starch to conceal its diluted condition, and it is even asserted that the clumsy fraud of adding chalk and emulsion of sheep's brains has been detected.

On examining a little of the milk under the microscope, the peculiar granules of starch and flour may be readily seen, larger and more oval than the milk globules, if either of those substances is present; and when examined with polarized light, each granule will be found to exhibit a dark cross. Should any doubt exist as to their real nature, the addition of a drop or two of a solution of iodine will impart to the farina granules a dark purple color.

Gum may be detected by acidulating the milk with acetic acid, boiling, filtering off the coagulum, and mixing the filtrate with alcohol, when the gum is deposited and may be recognized by its behavior with water. The presence of annatto would cause the milk to assume a brown color on addition of carbonate of soda.

The microscope will also serve to show the presence of macerated brain, which may be recognized by the occurrence of fragments of nerve and other organized structures, not found in pure milk.

The presence of chalk may be still more easily discovered, since, owing to its specific weight, it soon subsides to the bottom of the liquid, where it may at once be recognized by its effervescing on the addition of a little dilute hydrochloric acid.

We have no chemical means of ascertaining whether water has been fraudulently added to milk, the only effect being to dilute it, and render it of poorer quality, which might arise from natural causes. A knowledge of the specific gravity will not even allow us to decide as to the richness of the milk, since the abstraction of a portion of the cream, which has a lower specific gravity than milk, may be made to neutralize the effect produced by the addition of water; the tendency of the removal of the cream being to raise the specific gravity of the fluid, and that of the addition of water, to lower it. A specimen of milk, therefore, which has been impoverished by the abstraction of its cream, and still further weakened by the addition of water, may be made to possess the same specific gravity as it had when taken pure from the udder.

For most practical purposes it is sufficient to compare the relative volumes of cream furnished by equal quantities of different specimens of milk. This may be readily effected by allowing the milk to stand in a graduated tube *(lactometer)* for twenty-four hours, at a moderate temperature, and measuring the number of divisions occupied by the cream.

Another method proposed by Daubrawa for the rapid determination of the quality of milk consists in mixing it with two volumes of alcohol of sp. gr. 0·833, filtering off the butter and casein (which may be dried and weighed,) and taking the specific gravity of the filtrate. Every increase of ·004 in the specific gravity above 0·905 (the sp. gr. of the mixture of alcohol with the water of the milk) indicates 1 per cent. of milk-sugar.—For example, if the specific gravity of the filtrate be 0·922, there would be 4-25 per cent. of milk sugar, for 0-922—0-905=.017, and -017=-004=4-25. The result may be controlled by evaporating the spirit, converting the milk-sugar into grape-sugar by boiling with a little dilute sulphuric acid, rendering the solution alkaline by potash, and determining the sugar by the standard copper solution (352.)

It occasionally happens that the milk exposed for sale is the produce of an unhealthy animal. Such milk has usually some peculiarity of taste or smell, and also a slightly viscid and unnatural appearance; on being examined under the microscope, too, it will probably be found to contain pus or mucus corpuscles, or to present other appearances differing from those of the healthy secretion.

